# Influence of X-Chromosome Inactivation in Pathogenesis of Turner Syndrome

**DOI:** 10.3390/epigenomes10030043

**Published:** 2026-07-02

**Authors:** Ana-Maria Grigore, Lavinia Caba, Vlad Teodor Iacob, Lucian-Mihai Antoci, Monica Cristina Pânzaru, Lăcrămioara Ionela Butnariu, Eusebiu Vlad Gorduza

**Affiliations:** 1 Grigore T. Popa University of Medicine and Pharmacy, 700115 Iasi, Romania; grigore_ana-maria@d.umfiasi.ro (A.-M.G.); vlad-teodor.iacob@umfiasi.ro (V.T.I.); lucian-mihai.antoci@umfiasi.ro (L.-M.A.); monica.panzaru@umfiasi.ro (M.C.P.); ionela.butnariu@umfiasi.ro (L.I.B.); eusebiu.gorduza@umfiasi.ro (E.V.G.); 2 Mother and Child Medicine Department, Faculty of Medicine, Grigore T. Popa University of Medicine and Pharmacy, 700115 Iasi, Romania; 3 Medical III Department, Faculty of Medicine, Grigore T. Popa University of Medicine and Pharmacy, 700115 Iasi, Romania

**Keywords:** X-chromosome inactivation, Turner syndrome, monosomy X, epigenomics

## Abstract

Turner syndrome (TS), a disorder caused by the complete or partial absence of an X chromosome, exhibits significant clinical variability that cannot be fully explained by chromosomal anomalies alone. This narrative review highlights the crucial role of epigenetic mechanisms, particularly X-chromosome inactivation (XCI), in shaping the TS phenotype. The haploinsufficiency of genes that normally escape XCI is a primary driver of TS features. The specific epigenetic consequences depend on the chromosomal anomaly. In complete monosomy (45,X), the absence of escape-mediated dosage compensation genes from a second X chromosome amplifies haploinsufficiency across X-linked escape genes. Isochromosome Xq (i(Xq)) variants involve the loss of the short arm (Xp) and duplication of the long arm (Xq), creating a dual dosage imbalance with extreme XCI skewing. Carriers of i(Xq) also have a heightened risk for autoimmune disorders compared to those with 45,X TS. For ring-X chromosomes (r(X)), which are mitotically unstable, the functional status of the *XIST* gene is critical. If the ring is *XIST*-negative, it remains transcriptionally active, resulting in functional disomy and a more severe phenotype with pronounced neurodevelopmental and craniofacial features. Ultimately, the clinical heterogeneity in TS arises from a complex interplay of the specific chromosomal structure, tissue-specific mosaicism, *XIST* function, and variable escape from XCI, defining TS as a disorder of epigenetic and gene-regulatory imbalance. However, future research requires a better understanding of the complex mechanism of X-chromosome inactivation.

## 1. Introduction

Turner syndrome (TS) is the most frequent disease that causes genetic female infertility, and it occurs in about 1/2500 female newborns. Complete or partial X monosomy causes this disorder, being the only monosomy that allows for the survival of a human embryo. The main clinical features are short stature and streak ovaries, primary ovarian insufficiency, primary amenorrhea and deficiency of female secondary sexual characteristics. Clinical diagnosis requires confirmation by a karyotype [1,2].

The model of X-chromosome inactivation (XCI), proposed by Mary Lyon in 1961, presupposes a stable silencing of genes on one X chromosome in female somatic cells [3]. This is an epigenetic process essential for biological equalization between female and male organisms in terms of the activity of genes located on the X chromosome [4]. In reality, this silencing is incomplete, and a defined subset of genes remains expressed on the inactive X, creating a dosage contribution that is biologically meaningful and, in several cases, clinically critical [4,5,6].

This dosage mechanism is particularly relevant in Turner syndrome (TS) because many of its clinical features are best understood not simply as the direct consequence of missing a chromosome but as the result of an insufficient dosage of X-linked genes that normally require two active copies [7,8]. Genes that typically escape XCI are expressed by both X chromosomes in 46,XX individuals, but their expression drops to a single dose when only one X chromosome is present, like in females with Turner syndrome and complete X monosomy (45,X). An example is the *SHOX* (short-stature homeobox) gene, located in the PAR1 region, whose haploinsufficiency generates short stature and characteristic skeletal findings [9,10,11]. However, this reduced dosage seems to contribute to gonadal dysgenesis, cardiovascular anomalies and other recurrent TS traits [12].

Clinical variability in TS directly reflects the underlying variation in X-chromosome constitution. The 45,X karyotype is the main chromosomal formula found in TS, but many individuals present mosaicism or unbalanced structural abnormalities, such as Xq isochromosomes, ring X chromosomes or segmental deletions [13,14]. Thus, both the specific gene content and epigenetic dosage regulation modulate the TS phenotype. Furthermore, clinical outcomes depend on several factors, such as the existence of normal or modified silencing by cellular selection and the loci that are subject to silencing [15,16]. A good example in this respect is the ring X chromosome. If the ring retains an intact X-inactivation center (including the *XIST* gene—X inactive specific transcript), it can undergo inactivation, and the individual presents with a typical TS phenotype [17]. If *XIST* is absent, the X ring may remain transcriptionally active, driving abnormal dosage with significantly more severe developmental outcomes [18].

This review examines how different unbalanced structural abnormalities of X chromosomes and XCI behavior contribute to the clinical heterogeneity observed across Turner syndrome, focusing on both complete X monosomy and incomplete X monosomy (X isochromosome and X ring chromosomes).

## 2. Origins and Mechanisms of Chromosomal Anomalies in Turner Syndrome

Chromosomal anomalies that result in the TS phenotype are summarized in Table 1. The mechanisms that generate these chromosomal anomalies are heterogenous [13]. Understanding how each karyotype arises is essential for interpreting its downstream dosage consequences.

Complete monosomy X (45,X) typically results from nondisjunction, premature chromatid separation during parental gametogenesis, or postzygotic mitotic loss of one sex chromosome in an early embryonic cell [35]. Molecular haplotyping studies indicate that in approximately 60–80% of cases, the retained X has maternal origin, with an error having occurred during paternal meiosis II or, less frequently, during early mitotic divisions [35,36]. In TS cases with 45,X, a viability paradox is present. Despite the relatively low severity of the phenotype, over 99% of 45,X conceptions are lost spontaneously [37,38]. This could be explained by the rescue hypothesis, which states that many live-born individuals diagnosed as non-mosaic 45,X, in fact, carry a cryptic euploid (46,XX or 46,XY) cell lineage in extraembryonic tissues that sustains embryogenesis [37,39].

Isochromosome Xq (i(Xq)) most commonly arises through U-type sister–chromatid exchanges via postzygotic mitotic or meiotic mechanisms, producing a metacentric chromosome with two identical Xq arms and the complete absence of Xp. Parental origin studies have indicated roughly equal maternal and paternal contributions [35,40]. Because the underlying event is frequently postzygotic, the resulting 45,X/46,X,i(Xq) constitution is often mosaic, with clone proportions subsequently shaped by cellular selection [41].

Ring X chromosomes form via telomere–telomere fusion or end-to-end ring closure, sometimes following an interstitial deletion and DNA repair-mediated rearrangement [42,43]. Retained euchromatic content and, thus, phenotypic severity depend critically on breakpoint positions [44]. Other structural variants (terminal Xp and Xq deletions and X autosome translocations) arise through analogous recombination and repair mechanisms, with phenotypic consequences governed by the specific gene content that has been gained or lost [30,45].

## 3. Initiation of X-Chromosome Inactivation (XCI) and the XIC/XIST Axis

**Figure 1:**
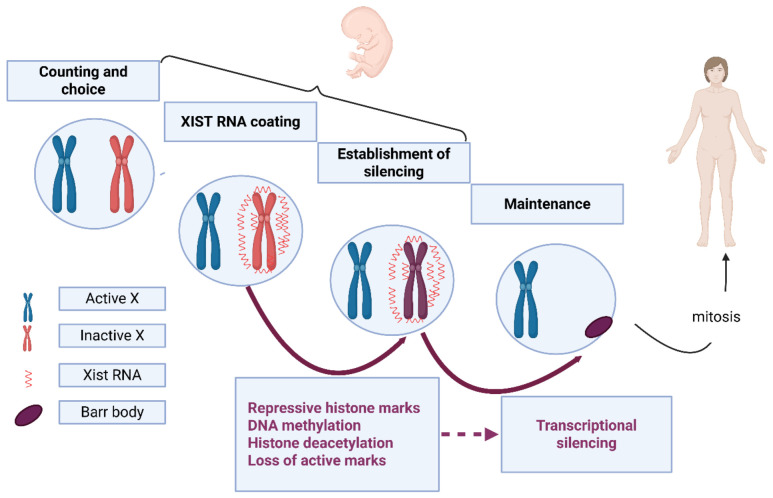
X-chromosome inactivation: from initiation to maintenance. Created in BioRender. Caba, L. (2026) https://BioRender.com/1w8j6vs (accessed on 27 April 2026).

X-chromosome inactivation (XCI) goes through four sequential phases: counting and choice; XIST RNA coating; establishment of silencing; and maintenance of the inactive state through mitosis, resulting in stable transcriptional silencing (Figure 1). This process starts in the early stages of mammalian embryogenesis under the control of the X-inactivation center (named XIC in humans/Xic in mammals) [46,47].

This center is located at Xq13.2 and represents a ~1 Mb cis-regulatory hub located on the human X chromosome [48,49]. The XIC constitutes the master regulatory locus for XCI and houses XIST, together with a constellation of cis- and trans-acting noncoding RNAs and regulatory elements that collectively tune Xist expression and bias the choice of the future inactive X (Xi) [50].

The *XIST* gene (X Inactive Specific Transcript) is approximately 32 kb in humans and is transcribed exclusively from the Xi allele. Its pre-mRNA is processed into a ~19 kb spliced, polyadenylated lncRNA that is not translated into protein [51]. The *XIST* transcript contains several functionally distinct repeat domains (Repeats A–F). Repeat A recruits SPEN/HDAC3 for transcriptional silencing and mediates nuclear lamina tethering through LBR. Repeats B/C recruit PRC1/PRC2 via hnRNPK, repeat E drives phase-separated compartment formation, enabling the spread of chromosome-wide silencing, and repeat F is required for the distal propagation of transcriptional silencing [52,53,54,55].

In murine models, the *Xic* contains multiple noncoding regulators (e.g., *Ftx*, *Xite*, and *Tsix*) and protein-coding regulators (e.g., RNF12/RLIM) that form regulatory loops balancing the activation and repression of Xist, thereby influencing counting and choice [56]. Notably, RNF12 acts as a trans-acting activator whose dosage helps overcome *Tsix* repression and biases initiation toward XCI when more than one X chromosome is present [57,58].

Mechanistic models increasingly emphasize that counting relies on a dosage- and threshold-sensitive network of X-linked activators and autosomal/cis repressors rather than a simple “two XICs are required” rule [59,60]. Trans-acting factors outside the XIC can compensate when parts of the XIC are deleted, and XIC–XIC pairing is not strictly required for monoallelic *Xist* activation, though transient pairing may modulate timing or efficiency in certain cellular contexts [50,61]. In human development, on the intact X, XCI initiation can proceed even when *XIST* is missing from the deleted chromosome in some cases. This highlights the influence of trans-acting activators outside the deleted region and signals conservation of the regulatory logic across mammals [49]. XCI initiation starts at a developmental time point and requires a multi-layered network rather than an absolute, binary requirement for dual XICs [62].

Following initiation, the spread of silencing occurs as XIST-coated chromatin recruits polycomb repressive complexes (PRC2 and PRC1) [55,63]. PRC2 and PRC1 work together via H3K27me3 and H2AK119ub, respectively, to transform the genetic material of the X chromosome into X chromatin by globally reducing transcription at the level of the inactivated X chromosome [64,65]. This spreading requires changes in nuclear organization and chromosome topology [66]. A cis-acting XIC hub allows for XCI through the intervention of a trans-acting regulatory network and chromatin modifiers that establish and maintain Xi, while escape genes, 3D genome architecture and lineage context shape the quantitative outcomes of dosage compensation [49,67]. The maintenance of this repressive state across cell divisions relies on additional layers of epigenetic regulation. Promoter DNA methylation on Xi and other chromatin regulators such as *SMCHD1* generate constant compaction and suppress reactivation, though Xi maintenance can be context-dependent [68,69]. In some cases, inactivation may be reversible, like in pluripotent cells or during reprogramming, reflecting the intrinsic epigenetic plasticity of Xi [52,70,71].

**Figure 2:**
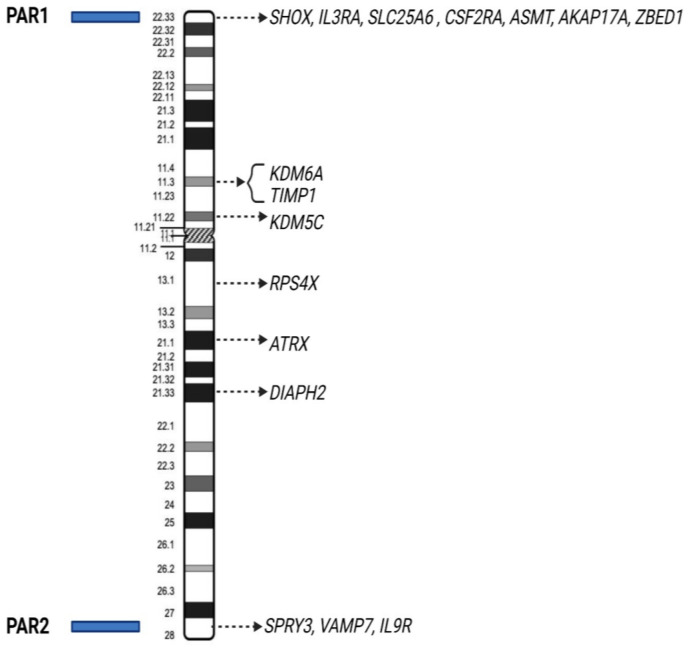
Selected escape genes. Adapted from ISCN 2024 [91]. Created in BioRender. Caba, L. (2026) https://BioRender.com/s58zptg (accessed on 3 June 2026).

In humans, approximately 15–23% of X-linked genes escape XCI, especially in PAR regions (PAR1 and PAR2; PAR—pseudoautosomal region) but also evident outside PARs in tissue- and lineage-specific patterns [5]. Escape genes intervene in health and disease in both sexes and across developmental stages [72,73]. Examples of genes that escape X silencing are *SHOX* (located on PAR1) and the *KDM6A* and *KDM5C* genes (present in a non-PAR region), all of which ae associated with phenotypic variation in cases of X haploinsufficiency [74,75]. Random XCI generates cellular mosaicism, with neighboring cells expressing alternative maternal or paternal X-linked alleles in 46,XX females, whereas TS-related structural variants show extreme skewing, driven by post-inactivation cellular selection and tissue-specific mosaicism [76]. The principal escape genes implicated in the TS phenotype, their XCI status and the supporting evidence are summarized in Table 2 and Figure 2.

Critically, escape from XCI is not a fixed, constitutive property of individual loci but varies substantially across cell types and tissues [4,92]. Large-scale single-cell quantification using the scLinaX (single cell Lina X) framework applied to approximately one million blood cells demonstrated that escape is significantly stronger in lymphocytes than in myeloid cells at both the transcriptional and chromatin-accessibility levels, a finding replicated across multiple human-organ scRNA-seq datasets [93]. This cell-type specificity has direct implications for the interpretation of TS phenotypic data, the majority of which is derived from peripheral blood lymphocytes rather than the disease-relevant tissues of the heart, brain, or ovaries [72,93].

Across mammals, core XIST/XIC logic is conserved, but regulatory architecture, including that of long noncoding RNAs (lncRNAs); expression dynamics; and higher-order chromatin topology show lineage- and species-specific adaptations, highlighting the evolutionary flexibility of XCI control [94,95]. Mechanistic studies show a close connection between XCI and pluripotency networks; thus, the XCI trajectory in early development and in vitro pluripotent states is dynamic and subject to variation under specific culture and developmental conditions [96,97].

Escape from XCI is a widespread feature that shapes dosage-sensitive phenotypes [72,73]. The interplay between escape and Xi’s three-dimensional organization shapes our understanding of sex-biased disease susceptibility and developmental paths [98]. Turner syndrome-related mosaicism often arises from selection against cells with deleterious dosage imbalances, which leads to skewed XCI patterns that vary by tissue [99]. The presence of unbalanced structural anomalies of the X chromosome (like ring or isochromosome anomalies) favors the appearance of dynamic mosaicism due to mitotic instability, with implications for neurocognitive function and systemic health [16,30,42].

The choice of X-chromosome silencing and the conservation of the XIST/XIC axis are species-specific processes that depend on regulatory elements, lncRNA complements and topological organization. In placental mammals, the XIC-XIST axis remains central, with a mosaic of cis- and trans-acting regulators. Bipartite XIC topology and topologically associating domain (TAD)-level regulation emphasizes how three-dimensional genome architecture tunes Xist regulation and the onset of silencing. This process shows some cross-species features, like shared strategies, as well as some lineage-specific innovations (e.g., XACT in primates as a potential counter-regulator in pluripotent states) [100,101,102]. However, XCI presents with variability. Thus, Xi stability is characteristic of mature somatic cells but with possible erosion in certain pluripotent or culture conditions, underscoring the importance of the cellular state in interpreting XCI dynamics [52,70,103].

## 4. Complete X Monosomy

The complete X monosomy generates Turner syndrome (TS), one of the most frequently observed sex-chromosome aneuploidies among live-born females [104,105].

### 4.1. XCI Counting, XIST Activity and Haploinsufficiency in Single X-Bearing Cells

In monosomy X, the X-inactivation counting mechanism detects the absence of a supernumerary X; thus, XCI is not initiated. The single X chromosome remains transcriptionally active, lacking the compensatory layer provided by an inactive homolog. In contrast to 46,XX cells, where the inactive X contributes to a partial dosage boost via escape genes, monosomy X entails a complete absence of this escape-mediated dosage compensation, amplifying haploinsufficiency across X-linked escapees and regulatory loci [16,106]. The *SHOX* gene is present in two functional copies in 46,XX individuals, while a monoallelic expression in 45,X individuals generates skeletal anomalies such as short stature and Madelung deformity [107]. Beyond the *SHOX* gene, a broader network of escape genes (e.g., *KDM6A* and *KDM5C*) intervenes in the development of different anatomic components, and the absence of a second allele in TS is associated with infertility, neurocognitive development, and cardiac anomalies [79].

### 4.2. Imprinted Parent-of-Origin Effects and Phenotypic Consequences

Although TS karyotypes are cytogenetically indistinguishable in terms of paternal versus maternal X-chromosome loss, epigenetic imprinting could influence the expression of X-linked genes, contributing to neurodevelopmental and cardiovascular variability [108,109]. Evidence from clinical and experimental work suggests associations between maternal X retention and certain congenital anomalies, as well as attenuated or enhanced social–cognitive profiles depending on the parent of origin, though loci underpinning imprinting effects remain to be definitively identified [110,111]. Notably, in 60% to 80% of complete X monosomy, the retained X chromosome has maternal origin, and the aneuploidy is produced by paternal meiotic non-disjunction [13]. However, the clinical evidence for strong, consistent phenotypic differences based on parental origin remains inconclusive across studies [112]. The imprinting framework is consistent with broader observations that XCI interacts with early developmental programming and that imprinting dynamics can shape later disease risk in sex-chromosome aneuploidies [113,114].

On the epigenomic level, 45,X cells exhibit widespread DNA hypomethylation and altered higher-order chromatin organization [115]. These changes represent downstream consequences of the haploinsufficiency of X-linked regulatory genes rather than primary defects in the global epigenetic machinery [108]. The combination of haploinsufficiency and altered chromatin transforms Turner syndrome in a regulatory-imbalance disorder with far-reaching consequences for gene expression networks beyond the X chromosome and across multiple tissues [116]. Integration of transcriptomic and epigenomic data from early human development and TS patient-derived tissues emphasizes the global impact of monosomy X on developmental gene-regulatory circuits, including neural crest derivatives and cardiovascular pathways [117,118].

Turner syndrome is a multisystem condition with short stature and ovarian dysfunction as hallmark features, but cardiovascular anomalies (e.g., bicuspid aortic valve or aortic coarctation), renal malformations, lymphedema and skeletal anomalies are also prevalent [7,119]. Furthermore, there is an increased prevalence of autoimmune disorders such as Hashimoto’s thyroiditis and metabolic conditions like type 2 diabetes [112]. The phenotypic spectrum is wide and variable, reflecting both the extent of X-chromosome loss and tissue-specific sensitivity to haploinsufficient escape genes [107]. While the *SHOX* dosage largely accounts for the skeletal features, cardiovascular and metabolic phenotypes likely arise from the modest effects of multiple escape genes (like the *TIMP1* and *TIMP3* genes, which are risk factors for bicuspid aortic valves and aortopathy) in combination with non-escape X-linked genes, as well as autosomal modifiers and epigenetic context [13,112,120]. At the placental level, some immune-regulatory genes and metabolic pathways are disturbed in monosomy X and are associated with pregnancy outcomes [121].

Recent studies on dosage compensation have linked monosomy X biology to tissue-specific gene networks. Preclinical and patient-derived models (e.g., isogenic hiPSC (human induced pluripotent stem cells) panels) provide the necessary premises for understanding how X-chromosome gene-dosage perturbations affect neural-crest biology, cardiac ontogeny and endocrine development, with the aim of identifying candidate haploinsufficient drivers and potential therapeutic targets to mitigate TS-associated morbidity [106,117,122]. Comparative analyses across species have explained the principles of X-chromosome regulation, underscoring lineage-specific adaptations, with implications in basic biology and translational strategies for TS management [123,124].

## 5. Aneuploid Mosaic in Turner Syndrome

Mosaic TS without unbalanced X chromosome anomalies (e.g., 45,X/46,XX or 45,X/46,XY) is common and has implications for fertility, gonadal development and endocrine function [125,126]. Skewed XCI, mosaic cellular composition and the presence of X rearrangements modulate the penetrance and expressivity of TS phenotypes, including gonadal dysgenesis and reproductive potential [127]. The degree of mosaicism in 45,X/46,XX Turner syndrome directly impacts ovarian function, with a higher percentage of normal 46,XX cells correlating with a greater chance of spontaneous puberty. In some mosaic contexts, partial or complete rescue of dosage balance via cellular selection can occur, shaping long-term outcomes and contributing to variability in neurocognitive and cardiovascular care needs [128]. Thus, karyotype–phenotype relationships in TS are viewed as probabilistic rather than fixed, with tissue-specific mosaicism and lineage-restricted effects shaping the marked heterogeneity of clinical presentations [129,130].

## 6. Isochromosome Xq in Turner Syndrome

The 46,X,i(Xq) karyotype generates TS because of Xp monosomy [98]. This unique gene-dosage configuration sets up in opposition to the haploinsufficiency of some genes and three copies for other genes that shape the clinical phenotype during development, with tissue-specific consequences governed by both XCI patterns and escape-gene biology [131,132]. 46,X,i(Xq) is typically detectable by conventional cytogenetics or by FISH [133].

XCI skewing can guide interpretation, with the added complexity that i(Xq) may be present in a mosaic context across tissues [134]. XCI skewing and clonal selection i(Xq) cells show extreme XCI skewing, with the abnormal Xq-bearing chromosome preferentially silenced in surviving cells, particularly in peripheral blood lymphocytes [135]. In this case, post-inactivation clonal selection occurs, with preservation of Xp gene dosage while avoiding excess dosage on Xq to improve cellular viability [136]. However, XCI skewing can be tissue-specific, reflecting mosaicism and clonal dynamics across hematopoietic lineages and gonadal tissues that shape clinical outcomes [137].

The functional impact of i(Xq) reflects a complex dosage landscape. Haploinsufficiency for Xp loci coexists with overexpression of Xq material. The loss of *SHOX* from Xp contributes to growth impairment and skeletal anomalies common in i(Xq) carriers, mirroring aspects of 45,X monosomy but with added Xq content [102]. Meanwhile, Xq contains genes that escape XCI, resulting in functional Xq trisomy. Overexpression of select Xq escape genes (e.g., *RPS4X* and *DIAPH2*) may contribute to more subtle neurodevelopmental or learning profiles in some individuals, differentiating i(Xq) from pure 45,X genotype presentations [132,134].

Accurate delimitation of Xp loss and Xq duplication modifies prognosis and risk stratification for skeletal, gonadal, cardiovascular and autoimmune outcomes common in TS literature [138]. Ovarian dysgenesis and primary ovarian insufficiency remain notable in i(Xq) carriers, consistent with the essential role of Xp chromosome genes in ovarian development [139,140,141]. The paradox that i(Xq) patients often exhibit gonadal failure, despite the presence of three copies of Xq content, underlines that germ-cell survival depends on gene dosage, chromosomal context and XCI state [87]. Loss of Xp content disrupts germ-cell signaling and meiotic processes, while a duplicated Xq gene dosage—particularly among escapees—can further disrupt germ-cell development and maturation [86]. These dynamics highlight the need for vigilant reproductive counseling and endocrinology surveillance in i(Xq) TS patients [141].

Emerging clinical data suggest heightened autoimmune risk in i(Xq) carriers compared to 45,X TS, with autoimmune thyroiditis among the more frequently reported comorbidities in some series [142,143]. The gene-dosage framework proposes that Xq escape genes involved in immune regulation may contribute to dysregulated immune responses, whereas Xp haploinsufficiency could remove protective regulatory inputs [29]. The mechanistic basis remains under active investigation, with ongoing efforts to map how XCI escape and Xq overdosage influence immune-cell development and function in i(Xq) TS patients [144].

In the case of i(Xq), a unique chromatin architecture is modeled by epigenetics, chromatin context and three-dimensional genome features, generated by inter-relations between duplicated Xq content and the Xi regulatory network from the normal X chromosome [29]. XCI dynamics, escape-gene status and 3D genome organization induce the epigenetic state of the i(Xq) chromosome (fully silenced or variably active in specific tissues) [145]. Thus, epigenomic context contributes to the tissue-specific phenotypes and mosaic variation observed in i(Xq), guiding hypotheses about how structural architecture interacts with dosage-sensitive gene networks to shape the phenotype [76,146].

Given the dual effects of Xp haploinsufficiency and Xq overdosage, TS management in i(Xq) carriers requires multidisciplinary surveillance, including tracking of growth and gonadal function, as well as autoimmune screening and neurocognitive assessment, with attention to tissue-specific XCI patterns and mosaicism [132,147,148].

## 7. Ring X Chromosomes (46,X,r(X))

Ring X chromosomes—46,X,r(X)—represent a rare heterogeneous class of TS rearrangements [149,150]. In these cases, the karyotype from peripheral blood is not enough for the analysis of ring X burden, requiring multi-tissue cytogenetic assessment or integrative genomic approaches to delineate the ring spectrum and its phenotypic consequences [17,151].

A central determinant of the ring X phenotype is the retention or loss of the XIC including *XIST* at Xq13.2. *XIST*-positive rings retain a functional XIC, and *XIST* expression drives silencing of the ring and dosage normalization for retained genes [17,18]. Thus, patients have a TS phenotype, with severity shaped by the amount of euchromatic loss at breakpoints (including PAR1) [152].

In contrast, XIC and *XIST* are absent in *XIST*-negative rings, and the abnormal X chromosome remains transcriptionally active, resulting in functional disomy for ring content, producing Ring X syndrome with more pronounced neurodevelopmental and craniofacial features [153]. The XCI status of rings is frequently tissue-dependent, reflecting mosaicism and clonal dynamics across tissues and over development, consistent with broader TS XCI patterns in which *XIST* integrity and escape-gene dosage show tissue-specific effects [137,154].

Substantial phenotypic variability is present, even among *XIST*-positive rings, indicating that ring inactivation is not uniform across tissues. In particular, mosaic rings can show tissue-specific XCI spread, incomplete silencing or differential escape-gene activity. This can yield a broader phenotypic spectrum than a simple Turner-like phenotype would suggest [92,100,155]. Furthermore, in some cases, *XIST*-positive rings remain active in a subset of cells or tissues, contributing to functional disomy in those compartments despite the presence of *XIST* elsewhere [92,156]. Thus, a pathogenic mechanism linked only to XIST is unlikely. The mechanism is much more complex and includes XIST-dependent inactivation, tissue type, and the mode of cell-clone formation [157,158].

Clinical heterogeneity reflects the ring size, breakpoints and XCI status [137]. Dosage-sensitive Xp genes (e.g., *SHOX*) and Xq escape genes (e.g., *KDM5C*, *DIAPH2*, and *ATRX*) are plausible contributors to ring X phenotypes, with tissue context and mosaic ring representation shaping penetrance and expressivity across neurodevelopmental, endocrine and skeletal domains [159]. The literature supports a dosage-imbalance framework in Ring X syndrome, though gene-level contributions vary by case and require multi-tissue corroboration [160,161].

Neurodevelopment in Ring X syndrome reflects XCI status. *XIST*-positive rings often align with a Turner-like cognitive profile, attenuated by the ring’s euchromatic content and breakpoint positions [162,163]. *XIST*-negative rings commonly present more severe neurodevelopmental impairment, consistent with functional disomy of ring content and broader dosage imbalance affecting neural development [164]. Emerging multi-case ring X datasets emphasize that cognitive outcomes correlate with ring size, breakpoints and tissue mosaicism, underscoring the need for longitudinal neurodevelopmental assessment across tissues [43,165].

Ring X phenotypes frequently involve gonadal dysgenesis and ovarian dysfunction, particularly when Xp content is reduced or ring inactivation fails to normalize the dosage [32]. Skeletal anomalies and growth impairment are common in cases of Ring X syndrome. Both are correlated with dosage effects linked to Xp and Xq haploinsufficiencies and modulated by ring architecture and XCI status [166]. Ring X may disrupt meiotic pairing and germ-cell viability, complicating reproductive counseling and management and contributing to the Turner spectrum’s breadth with ring-specific variability [167].

## 8. Limitations and Gaps in Current Knowledge

In TS, karyotype–phenotype studies have touched on relatively small, clinically ascertained cohorts enriched for symptomatic individuals; thus, selection bias likely overstates phenotypic severity and may obscure the true prevalence and phenotypic range of mosaic forms, particularly low-level mosaicism that escapes standard karyotyping [23,168]. Compounding this, the majority of epigenomic and XCI data is derived from peripheral blood lymphocytes rather than clinically relevant tissues [108]. Given the well-documented tissue-specificity of XCI skewing and escape-gene expression, conclusions about XCI dynamics in the brain, heart, and ovaries remain inferential, awaiting validation in multi-tissue datasets [169,170].

Evidence for parent-of-origin effects on TS phenotypes is intriguing but remains mechanistically incomplete. X-linked loci subject to genomic imprinting have not been comprehensively mapped in humans, and functional studies on relevant TS model systems are lacking [110,111]. A related concern is cross-species extrapolation. Much of the mechanistic understanding of XCI counting, *XIST* regulation and escape-gene dynamics is derived from murine models whose regulatory architectures diverge substantially from the human context [123,171]. For instance, *XACT* (X-active coating transcript), which modulates *XIST* in primate pluripotent cells, has no murine ortholog, necessitating caution when translating murine findings to human TS biology [172].

At the resolution level, whole-genome bisulfite sequencing and ChIP-seq datasets for TS patient-derived tissues remain sparse, leaving the epigenomic landscape of structural X variants incompletely characterized [38,108]. Long-read sequencing technologies—notably, Oxford Nanopore—offer transformative potential for the phasing of methylation across large X-linked segments and the resolution of incomplete XCI in ring X and i(Xq) chromosomes, but large-scale application to TS cohorts is still emerging [106,173]. Finally, while the haploinsufficiency of several escape genes is well established (e.g., *SHOX* and *KDM6A*), the individual contributions of many candidate loci rest predominantly on correlative expression data. Thus, controlled functional experiments in isogenic human iPSC models could explain causality and quantify effect sizes [106,174].

Early Nanopore-based methylation profiling of 45,X patients has already begun to reveal a distinctive X-chromosome methylation spectrum in TS. Average methylation levels on the single X in monosomy more closely resemble those of 46,XY males than 46,XX females, a finding that directly implicates the absence of Xi-specific hypermethylation as a primary epigenomic driver in TS biology [175]. This early work demonstrates both the feasibility and diagnostic potential of long-read epigenomic approaches in TS and underscores the need for larger, multi-tissue Nanopore cohorts to resolve whether the observed methylation patterns are consistent across structural X variants [176,177].

## 9. Conclusions and Future Perspectives

In TS, various X-chromosomal configurations can be found. The complete X monosomy (45,X) is the common presentation, while structural variants such as isochromosome Xq (i(Xq)) and ring X (r(X)) substantially modify gene content and XCI dynamics, producing variable phenotypes. Many comprehensive studies emphasize the heterogeneity of karyotypes and their regulatory consequences, highlighting that phenotypes arise not only from numeric gene dosage but also from perturbations of regulatory networks, tissue-specific escape from XCI and the broader epigenetic context.

Structural X variants serve as informative natural models for dissecting the effects of gene dosage and regulation. Isochromosome Xq involves the loss of the short arm (Xp), with a duplication of the long arm (Xq), creating a scenario of haploinsufficiency for Xp genes and relative trisomy for Xq genes. This unique configuration shifts the dosage of PAR1 genes and other escape genes, with significant consequences for the TS phenotype. Ring chromosomes, on the other hand, are inherently unstable and can disrupt XCI silencing, leading to mosaicism and tissue-specific escape from inactivation. The clinical outcome in these cases often depends on the integrity of the X-inactivation center (XIC) and its critical component, *XIST*, which can diverge from expectations based on chromosomal content alone.

At the heart of TS pathology are the genes that escape XCI, particularly those located in PAR1. These genes are expressed by both X chromosomes in 46,XX individuals, so their dosage is halved in monosomy X, leading to haploinsufficiency. The *SHOX* gene in PAR1 is the clearest example; its reduced dosage is strongly linked to short stature and characteristic skeletal findings in TS. However, the classic TS phenotype—encompassing gonadal dysgenesis, cardiovascular anomalies, and renal malformations—results from the cumulative haploinsufficiency of multiple escape genes and broader regulatory-network perturbations, not just the *SHOX* gene. Reduced dosages of several X-linked genes that are essential for ovarian development and maintenance produce ovarian dysfunction.

These complexities underscore that diagnostic paradigms must extend beyond conventional karyotyping, which has limited prognostic resolution for structural variants. Detecting an i(Xq) or ring X chromosome should prompt targeted molecular characterization to assess *XIST* integrity, XCI status, and the specific euchromatic regions that have been retained or lost. Emerging epigenomic readouts, such as DNA methylation landscapes, integrated with long-read sequencing, provide a richer view of XCI maintenance and escape-gene dosage. Methodological advances, such as CRISPR–Cas9-based targeted enrichment, combined with long-read sequencing platforms like Oxford Nanopore, offer a powerful approach to map breakpoints, phase structural variations and assess native DNA methylation across large X-linked segments, enabling direct interrogation of incomplete XCI in complex variants.

This multi-omics approach can inform personalized risk stratification for TS-associated comorbidities like cardiovascular disease and primary ovarian insufficiency, guiding targeted surveillance and intervention strategies. The literature also highlights the influence of broader regulatory networks and tissue-specific escape patterns, particularly in mosaic or ring chromosome contexts. The precise contribution of non-PAR1 escape genes and the potential influence of imprinted genes and parental-origin effects on certain traits remain areas of active investigation, supporting a nuanced, multi-gene interaction model for the wide spectrum of Turner syndrome phenotypes.

## Figures and Tables

**Table 1:** Summary of principal Turner syndrome karyotypes and their consequences.

Karyotype	Approximate Frequency in TS	Key Genomic Consequence	Core Phenotypic Associations
45,X	40–50%	Complete X monosomy; no XCI; global haploinsufficiency of escape genes	Short stature, streak ovaries, primary amenorrhea, cardiovascular malformations, and lymphoedema [19,20,21]
45,X/46,XX	~15%	Mosaicism; degree of 45,X clone determines phenotype severity	Variable; may have spontaneous puberty or preserved fertility [22,23,24]
45,X/46,XY	~5–6%	Mosaicism with Y material; gonadoblastoma risk	Variable virilization; gonadal dysgenesis; gonadoblastoma risk ~15–20%; surveillance required [25,26,27]
46,X,i(Xq)	9–11%	Xp haploinsufficiency + Xq trisomy; extreme XCI skewing	Short stature and ovarian dysgenesis; heightened autoimmune risk vs. 45,X [20,28,29]
46,X,r(X) *XIST*+	~2–5%	Variable euchromatic loss; ring inactivated via *XIST*	Turner-like phenotype; severity correlates with breakpoint and PAR1 retention [17,30]
46,X,r(X) *XIST*-	Subset of r(X)	Functional disomy of ring content; no XCI on ring	Ring X syndrome: severe neurodevelopmental impairment and craniofacial anomalies [18,31]
46,X,del(Xp) or 46,X,del(Xq)	Rare	Partial deletion; gene content determines phenotype	Varies by segment; deletion of *SHOX* gene: short stature; Xq del: ovarian insufficiency [32,33,34]

**Table 2:** Selected escape genes implicated in the Turner syndrome phenotype.

Gene	Location	XCI Status	Phenotypic Consequence(s) in TS	Evidence Level
*SHOX*	PAR1 (Xp22.33/Yp11.32)	Escapes XCI (both X and Y)	Short stature, Madelung deformity, and skeletal dysplasia	Human genetics; expression studies; mouse models [77,78]
*KDM6A*	Xp11.3 (non-PAR)	Escapes XCI	Neurodevelopmental delay; Kabuki syndrome-like features	Human genetics; functional studies [79,80,81]
*KDM5C*	Xp11.22 (non-PAR)	Escapes XCI	Intellectual disability; sex-biased neurological vulnerability	Expression studies; model systems [79,82,83]
*RPS4X*	Xq13.1 (non-PAR)	Partial escape	Potentially modifies neurocognitive profile in i(Xq) TS	Expression studies [16,84,85]
*DIAPH2*	Xq21.33 (non-PAR)	Variable/uncertain XCI escape status	Ovarian development; gonadal dysgenesis in i(Xq) context	Human genetics; expression data [86,87]
*TIMP1*	Xp11.3 (non-PAR)	Variable/may partially escape	Implicated in connective tissue biology; potential contributor to aortopathy risk	Association studies; expression data [38,88,89]
*ATRX*	Xq21.1 (non-PAR)	Subject to XCI	Intellectual disability in *XIST*-negative ring X (functional disomy)	Case reports; functional studies [18,90]

## Data Availability

No new data were created or analyzed in this review article. Data sharing is not applicable to this article.

## Abbreviations

In this manuscript, we use the following abbreviations:

TS	Turner syndrome
XCI X	Chromosome inactivation XCI
i(Xq)	Isochromosome Xq
r(X)	Ring X chromosome
XIST	X inactive specific transcript
XIC X	Inactivation center
PAR1	Pseudoautosomal Region 1
PAR2	Pseudoautosomal Region 2
PRC1	Polycomb Repressive Complex 1
PRC2	Polycomb Repressive Complex 2
lncRNA	Long noncoding RNA
scLinaX	Single-cell LinaX
hiPSC	Human induced pluripotent stem cell
